# Complementary effects of phosphorus supply and planting density on maize growth and phosphorus use efficiency

**DOI:** 10.3389/fpls.2022.983788

**Published:** 2022-09-26

**Authors:** Haiqing Gong, Yue Xiang, Bilisuma Kabeto Wako, Xiaoqiang Jiao

**Affiliations:** National Academy of Agriculture Green Development, Department of Plant Nutrition, Key Laboratory of Plant-Soil Interactions, Ministry of Education, China Agricultural University, Beijing, China

**Keywords:** maize, phosphorus supply, planting density, phosphorus use efficiency, root morphological traits, soil P dynamics

## Abstract

Phosphorus (P) supply and planting density regulate plant growth by altering root morphological traits and soil P dynamics. However, the compensatory effects of P supply and planting density on maize (*Zea mays* L.) growth and P use efficiency remain unknown. In this study, we conducted pot experiments of approximately 60 days to determine the effect of P supply, i.e., no P (CK), single superphosphate (SSP), and monoammonium phosphate (MAP), and different planting densities (low: two plants per pot; and high: four plants per pot) on maize growth. A similar shoot biomass accumulation was observed at high planting density under CK treatment (91.5 g plot^–1^) and low planting density under SSP treatment (94.3 g plot^–1^), with similar trends in P uptake, root morphological traits, and arbuscular mycorrhizal colonization. There was no significant difference in shoot biomass between high planting density under SSP (107.3 g plot^–1^) and low planting density under MAP (105.2 g plot^–1^); the corresponding P uptake, root growth, and P fraction in the soil showed the same trend. These results suggest that improved P supply could compensate for the limitations of low planting density by regulating the interaction between root morphological traits and soil P dynamics. Furthermore, under the same P supply, the limitations of low planting density could be compensated for by substituting MAP for SSP. Our results indicate that maize growth and P use efficiency could be improved by harnessing the compensatory effects of P supply and planting density to alter root plasticity and soil P dynamics.

## Introduction

Phosphorus (P) is an essential nutrient for plant growth and development, as well as an important factor limiting sustainable maize production (Bindraban et al., 2020; Muhaba and Dakora, 2020). Mineral P fertilizers are critical in ensuring food security (Nedelciu et al., 2020); however, P use efficiency is extremely low owing to poor P mobility (Heuer et al., 2017). Although increasing planting density is a common agronomic measure for increasing P use efficiency (Jia et al., 2018; Zhang et al., 2021), it results in reduced space for root growth. The primary mechanism for P acquisition in maize production is the morphological expansion of root systems (Zhou et al., 2019a). The low mobility of P and the compression of root space have made efficient P use under high planting density conditions difficult (Shao et al., 2018). Allowing root systems to fully develop their biological potential and increase soil P uptake will be critical to improving P use efficiency in intensive maize production.

The spatial expansion of maize roots becomes severely compressed and the competition among plant roots growing closely at high planting densities is severe due to the extent of overlap among rhizospheres, which may result in nutrient deficiencies (Thorup-Kristensen and Kirkegaard, 2016; Li et al., 2019). High planting densities decrease the number of nodal roots, lateral root density, and root biomass per unit volume of soil (Shao et al., 2018; Niu et al., 2020). In addition, root system size can be reduced at high planting density due to plant competition. The root crown angle can become more vertical to adapt to a small growing space at high planting densities (Shao et al., 2018; Tomasi et al., 2020). Furthermore, maize roots adapt to obtain P in small spaces by becoming thinner and longer, extending into soil spaces with limited carbohydrates. The root-soil contact area also increases to enhance P acquisition (Lynch et al., 2022). Root foraging for P is determined by changes in root morphology in response to soil environmental conditions, and plant productivity and P uptake may be affected by planting density due to differential competitiveness.

The plastic response of the root system is not only limited by growth area but also largely affected by P supply at high planting densities (Wang et al., 2020; Postma et al., 2021). According to Semchenko (2020), resource capture is the primary driver of root changes. Maintaining an adequate P supply in the root zone can help the root system and mycorrhizal infection expand into the soil space and acquire soil P (Deng et al., 2017). Planting density and P supply interactions can maximize the efficiency of maize roots in mobilizing and acquiring P (Schneider et al., 2019). Indeed, P supply has the potential to considerably improve P use efficiency in crop production by activating soil P (Soltangheisi et al., 2018). Initial and residual P effects are influenced by the solubility of P fertilizer, which varies widely (Gong et al., 2022). However, it is unclear whether maize dry-matter accumulation loss caused by low planting density may be compensated for by regulating root plasticity and soil P dynamics through altered P supply.

This study aims to determine the compensatory mechanisms of P supply and planting density on maize root morphology, soil P dynamics, and P uptake. The specific goals of the study were to (1) evaluate the growth and P uptake of maize under different P supplies and planting densities, and (2) to examine whether maize growth limitation due to low planting density may be compensated for by altering P supply.

## Materials and methods

### Experimental design

A greenhouse pot experiment was conducted from May to July 2021 at the Quzhou Experimental Station of the China Agricultural University (36°51′57″N, 150°0′37″E), in Quzhou County, Heibei Province, China. Calcareous loam soil was collected from the Quzhou Long-Term Fertilizer Station (36°52′21″N, 115°03′15″E) of the China Agricultural University. The soil was air-dried and then sieved to pass through a 2-mm mesh. The soil type is calcareous fluvo-aquic soil from the North China Plain. The basic physical and chemical properties of the soil are as follows: pH value, 8.0; soil organic carbon content, 12.5 g kg^–1^; total nitrogen content, 1.3 g kg^–1^; soil Olsen-P content, 4.3 mg kg^–1^; and soil available potassium content, 194 mg kg^–1^. The soil properties were measured using the methods specified by Bao (2001).

The experiment was laid out in a 3×2 complete factorial design. Three P supply treatments were used: no P (CK), single superphosphate (SSP), and monoammonium phosphate (MAP). Two planting densities were tested: low (two plants per pot) and high (four plants per pot). Each of the six treatment combinations were replicated four times for a total of 24 pots. Each pot was 30 cm in diameter and 40 cm in height. A treatment rate of 150 kg P ha^–1^ was applied using the different P supplies, SSP and MAP. In the fertigation treatments, urea and muriate of potash were used as sources of nitrogen and potassium, respectively. Both nitrogen and potassium were applied at a rate of 150 kg ha^–1^. All fertilizers were incorporated and completely mixed with soil at planting.

Maize Zhengdan 958 seeds was used in this study; they were surface sterilized in 10% (v/v) H_2_O_2_ for 0.5 h, washed with deionized water, a supersaturated solution of calcium sulfate (CaSO_4_) was soaked for 1 day, and germinated in a dish with an aerated cover with wet filter paper for 3 days at 25 °C. Pots were watered daily to field capacity (18%, w/w) by watering every day. Temperatures ranged from a minimum of 20 °C at night to a maximum of 30 °C during the day.

### Plant harvest and sample analysis

The plants were harvested 60 days after planting. Shoots and roots were separated and shoots were first oven-dried at 105 °C for 0.5 h and then dried at 75 °C for 4 days to constant weight. The dry samples were weighed, crushed, and homogenized. Shoot P concentration was determined according to the standard vanadomolybdate method (Murphy and Riley 1962).

The root samples were carefully and thoroughly washed in a large volume of deionized water and then scanned using the EPSON root scanning software (Epson Expression 1600 pro, Model EU-35; Epson, Tokyo, Japan) at 400 points per inch. The WinRHIZO software (Pro2004b, version 5.0; Regent Instruments Inc., QC, Canada) was used to measure total root length. Lateral root density was calculated by dividing the number of lateral roots per 5 cm of the primary root by the 5-cm length of the primary root (Wu et al., 2019). Arbuscular mycorrhizal colonization analysis was performed according to the method of Trouvelot et al. (1986). Fine roots were cut into 1-cm-long segments and thoroughly mixed. Root samples were cleared with 10% (w/v) potassium hydroxide (KOH) at 90 °C for 2 h, and mycorrhizal colonization was stained using trypan blue. All roots were dried at 75 °C for 3 days and then weighed to calculate root dry biomass. The root/shoot ratio was defined as the ratio of root dry mass to that of the shoots (Marziliano et al., 2015).

Rhizosphere soil was taken from a 0–2-mm distance around the roots and collected by clearing the roots of debris; the rhizosphere soil was the loosely attached soil particles that were removed from around the roots (Cui et al., 2020; Zhou et al., 2022). Soil samples were then air-dried and passed through a 2-mm mesh sieve. Soil P fractions were analyzed sequentially and extracted using the Hedley method (Hedley et al., 1982). Soluble P is extractable with water and NaHCO_3_ (water-extractable P and NaHCO_3_-extractable P), which is considered labile P, and are immediately available to plants. Moderately labile P is extractable with NaOH (NaOH-extractable P), which is available to plants. Recalcitrant forms, such as HCl-extractable P and residual P, are considered non-labile P and are only available to plants over long time periods (Cross and Schlessinger, 1995).

### Statistical analysis

A two-way analysis of variance (ANOVA) test was used to examine the effects of P supply, planting density and their interacting effects on variables. All statistical analyses were performed using the SPSS statistical software (SPSS 20.0; SPSS Inc., Chicago, IL, USA). Significant differences among means were separated by the least significant difference test at the *P* ≤ 0.05 probability level. The graphs were constructed using Sigmaplot (version 10.0; Systat Software Inc., San Jose, CA, USA).

## Results

### Shoot biomass and P content

**Figure 1:**
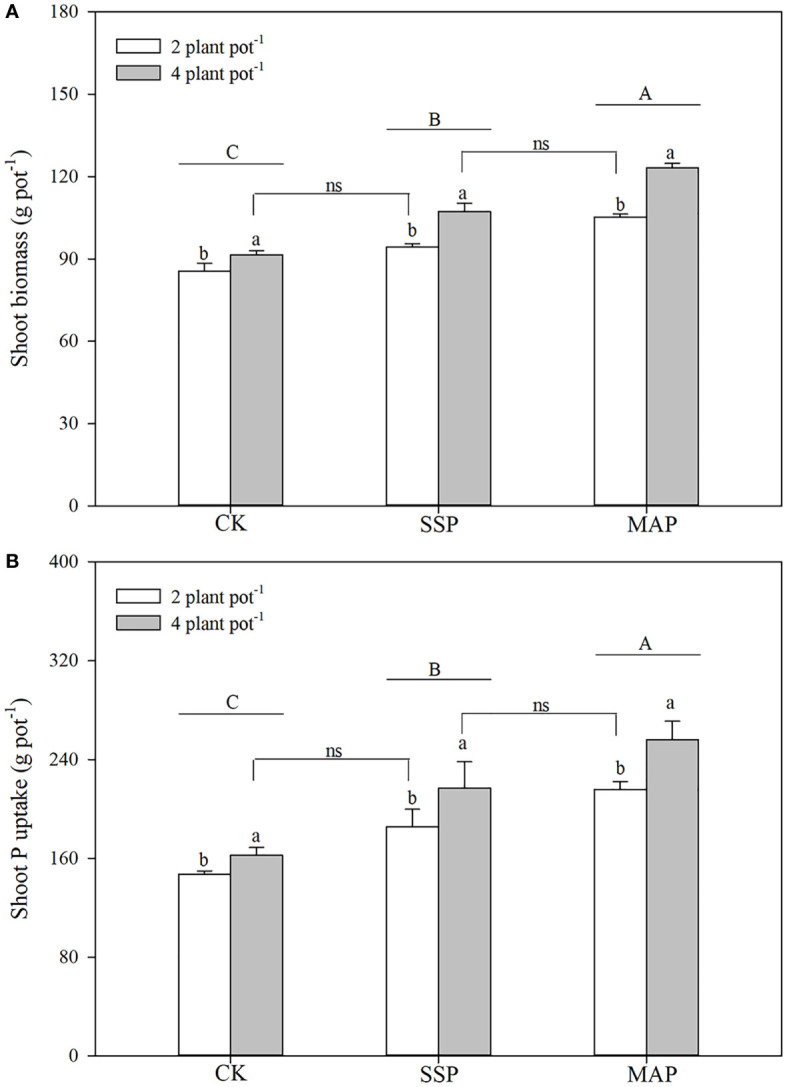
Shoot biomass **(A)**, shoot phosphorus (P) content **(B)** under two planting densities and three P fertilizer sources, no P (CK), superphosphate (SSP), and monoammonium phosphate (MAP). Different lowercase letters represent a significant difference among different planting densities at the *P* < 0.05 level, and different uppercase letters represent significant differences among different P fertilizer sources at the *P* < 0.05 level; ns indicates no significant difference at the 95% confidence interval between the two treatments.

Shoot biomass was significantly influenced by P supply and planting density (*P* < 0.01, **Table 1**; **Figure 1A**), and was higher for MAP-treated plants than for SSP or CK-treated plants at both planting densities. The high planting density concurrently improved shoot biomass: high shoot biomass was obtained under the high planting density condition, regardless of the P source. However, soil P supply intensity and planting density had complementary effects on shoot biomass. P supply intensity compensated for the reduction in shoot biomass due to the low planting density. Similar root biomass levels were recorded at the low planting density under the SSP treatment (91.5 g plot^–1^) and at the high planting density under the CK treatment (94.3 g plot^–1^). A strong complementary effect was also found between P source and planting density. A reduction in shoot biomass due to low planting density was compensated for by substituting the MAP for the SSP treatment under the same P supply. Higher shoot biomass was recorded for the high planting density under the SSP treatment (107.3 g plot^–1^), which was equivalent to the shoot biomass recorded for the low planting density under the MAP treatment (105.2 g plot^–1^).

**Table 1:** Two-way analysis of variance of phosphorus (P) supply, planting density, and their interaction.

Parameters	P supply		Planting density		P supply × Planting density	
	*F*-value	*P*-value	*F*-value	*P*-value	*F*-value	*P*-value
Shoot biomass	254.0	*P* < 0.0001	175.3	*P* < 0.0001	13.9	*P* = 0.0008
Shoot P uptake	61.1	*P* < 0.0001	23.2	*P* = 0.0004	1.5	*P* = 0.2655
Root biomass	71.5	*P* < 0.0001	72.7	*P* < 0.0001	2.5	*P* = 0.1213
Root length	25.7	*P* = 0.0003	46.4	*P* < 0.0001	0.5	*P* = 0.4785
Root/shoot ratio	12.3	*P* = 0.0044	6.9	*P* = 0.0097	3.0	*P* = 0.0875
Lateral root density	30.0	*P* < 0.0001	238.2	*P* < 0.0001	5.8	*P* = 0.0116
Labile P	5.3	*P* = 0.0407	2232.0	*P* < 0.0001	30.1	*P* < 0.0001
Moderately labile P	10.8	*P* = 0.0064	144.8	*P* < 0.0001	2.9	*P* = 0.0969
Non-labile P	2.1	*P* = 0.1738	9.2	P = 0.0039	2.2	*P* = 0.1555
AM colonization	21.7	*P* = 0.0002	278.2	*P* < 0.0001	0.4	*P* = 0.6815

AM, arbuscular mycorrhizal.

Shoot P content was strongly influenced by both P supply (*P* < 0.01) and planting density (*P* < 0.01), increasing in tandem with planting density (**Table 1**; **Figure 1B**). Shoot P content was higher in high planting densities than in low planting densities. The shoot P content observed in the low planting density under the SSP treatment was equivalent to the shoot P content observed in the high plant density under the CK treatment. This finding indicated that, although the shoot P content was limited by the low planting density, it was compensated for by P supply intensity. The shoot P content differed significantly by P source; regardless of planting density, the shoot P content was significantly higher when the MAP treatment was applied than when SSP was applied. Similar complementary effects of P sources and planting density on the shoot P content were observed. There were no significant differences in the shoot P content between the high planting density under the SSP treatment and the low planting density under the MAP treatment.

### Root morphological traits

**Figure 2:**
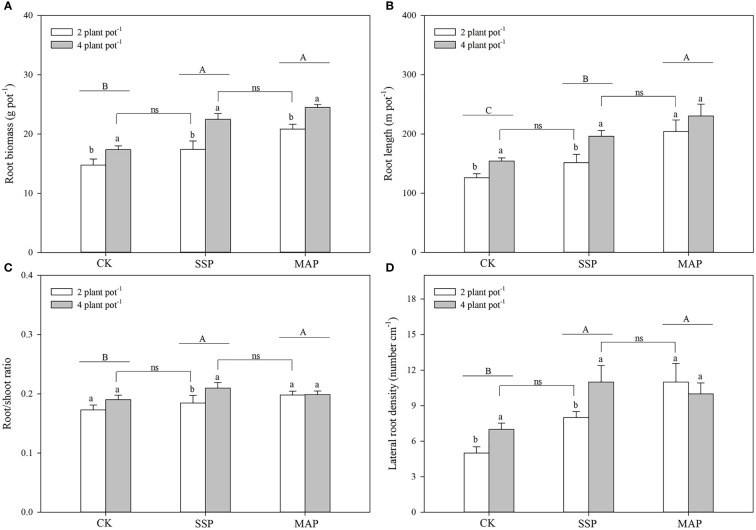
Maize root biomass **(A)**, root length **(B)**, root/shoot ratio **(C)**, and lateral root density **(D)** under two planting densities and three P fertilizer sources, no P (CK), superphosphate (SSP), and monoammonium phosphate (MAP). Different lowercase letters represent a significant difference among different planting densities at the *P* < 0.05 level, and different uppercase letters represent significant differences among different P fertilizer sources at the *P* < 0.05 level; ns indicates no significant difference at the 95% confidence interval between the two treatments.

P supply and planting density significantly influenced maize root biomass (*P* < 0.01, **Table 1**; **Figure 2A**). P supply had a greater influence on root biomass in the MAP treatment than in the CK or SSP treatments, and root biomass increased as planting density increased. No significant differences were observed between the low planting density under the SSP treatment and the high planting density under the CK treatment. There was a strong complementary effect between P source and planting density on root biomass. A similar root biomass accumulation was observed at the high planting density in SSP-treated pots and the low planting density in MAP-treated pots.

Maize root length was also affected by P supply and planting density (*P* < 0.01, **Table 1**; **Figure 2B**); specifically, it was higher in the MAP-treated pots than in both the SSP- or CK-treated pots, regardless of the planting density. Furthermore, maize root length increased with planting density for both of these P sources. However, there was no significant difference in the root length between the low planting density under the SSP treatment and the high planting density under the CK treatment. Root length in SSP-treated pots at the high planting density was equivalent to that in MAP-treated pots at the low planting density.

The root/shoot ratio and lateral root density were significantly altered by P supply and planting density (*P* < 0.01, **Table 1**; **Figures 2C, D**). Thus, the root/shoot ratio and lateral root density of plants grown in SSP- and MAP-treated pots were significantly greater than those grown in CK-treated pots. However, there was no significant difference in the root/shoot ratio or lateral root density between the SSP and MAP treatments at either planting density. The effect of planting density on the root/shoot ratio and lateral root density under MAP supply was negligible. Similar to root length, there was no significant difference in the root/shoot ratio or lateral root density between the low planting density under SSP and the high planting density under the CK treatment. Similar root/shoot ratio values and lateral root densities were observed at the high planting density under SSP and the low planting density under the MAP treatment.

### Phosphorus fractions in the rhizosphere soil of maize

**Figure 3:**
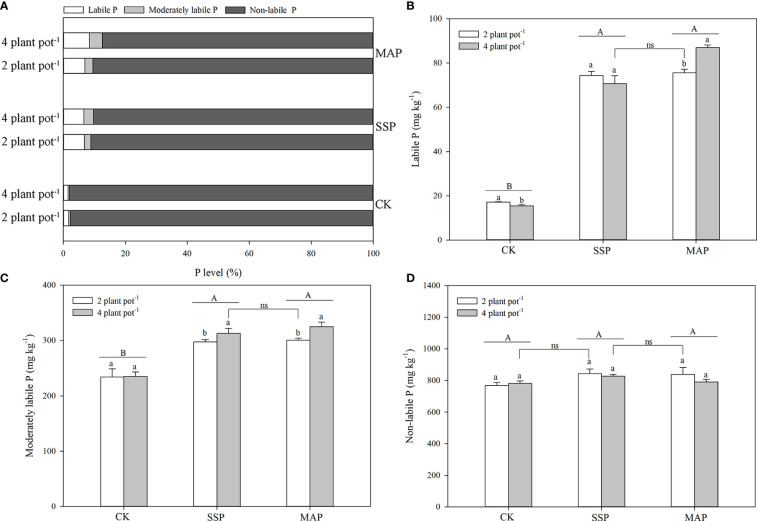
Proportions of labile, moderately labile, and non-labile P fractions under phosphate sources and cover crops under two planting densities and three P fertilizer sources **(A)**. Labile P **(B)**, moderately labile P **(C)**, and non-labile P **(D)** under two planting densities and three different P fertilizer sources, no P (CK), superphosphate (SSP), and monoammonium phosphate (MAP). Different lowercase letters represent a significant difference among different planting densities at the *P* < 0.05 level, and different uppercase letters represent significant differences among different P fertilizer sources at the *P* < 0.05 level; ns indicates no significant difference at the 95% confidence interval between the two treatments.

The labile P fractions accounted for only 2–8% of total P (**Figure 3A**). Labile P in the rhizosphere soil of maize was significantly altered by P supply and planting density (*P* < 0.01, **Table 1**; **Figure 3B**). The amount of labile P in maize plants grown with SSP or MAP was significantly greater than that of maize plants grown without P, while there was no significant difference in labile P between the SSP and MAP treatments, regardless of planting density. However, labile P in the rhizosphere soil of maize, in the high planting density under MAP was significantly greater than that of the low planting density treatment. There was no significant difference between the high planting density under the SSP treatment and the low planting density under the MAP treatment.

The moderately labile P fractions accounted for only 0.4–4% of total P (**Figure 3A**). Changes in the moderately labile P content of maize rhizosphere soil were mostly consistent with those for the labile P content among the treatments (**Figure 3C**). Moderately labile P in the maize rhizosphere soil significantly increased when P was supplied, compared with the CK treatment. A higher moderately labile P content in the maize rhizosphere soil was observed at the high planting density under the MAP treatment, whereas no significant difference was detected in moderately labile P between the high planting density under the SSP treatment and the low planting density under the MAP treatment.

The non-labile P fractions accounted for 88–98% of total P (**Figure 3A**). P supply and planting density had little effect on the non-labile P content in maize rhizosphere soil (**Table 1**; **Figure 3D**). However, the non-labile P content at the low planting density under the CK treatment was equivalent to that at the high planting density under the SSP treatment. Similarly, the non-labile P content was higher at the high planting density under the SSP treatment than at the low planting density under the MAP treatment.

### Arbuscular mycorrhizal colonization

**Figure 4:**
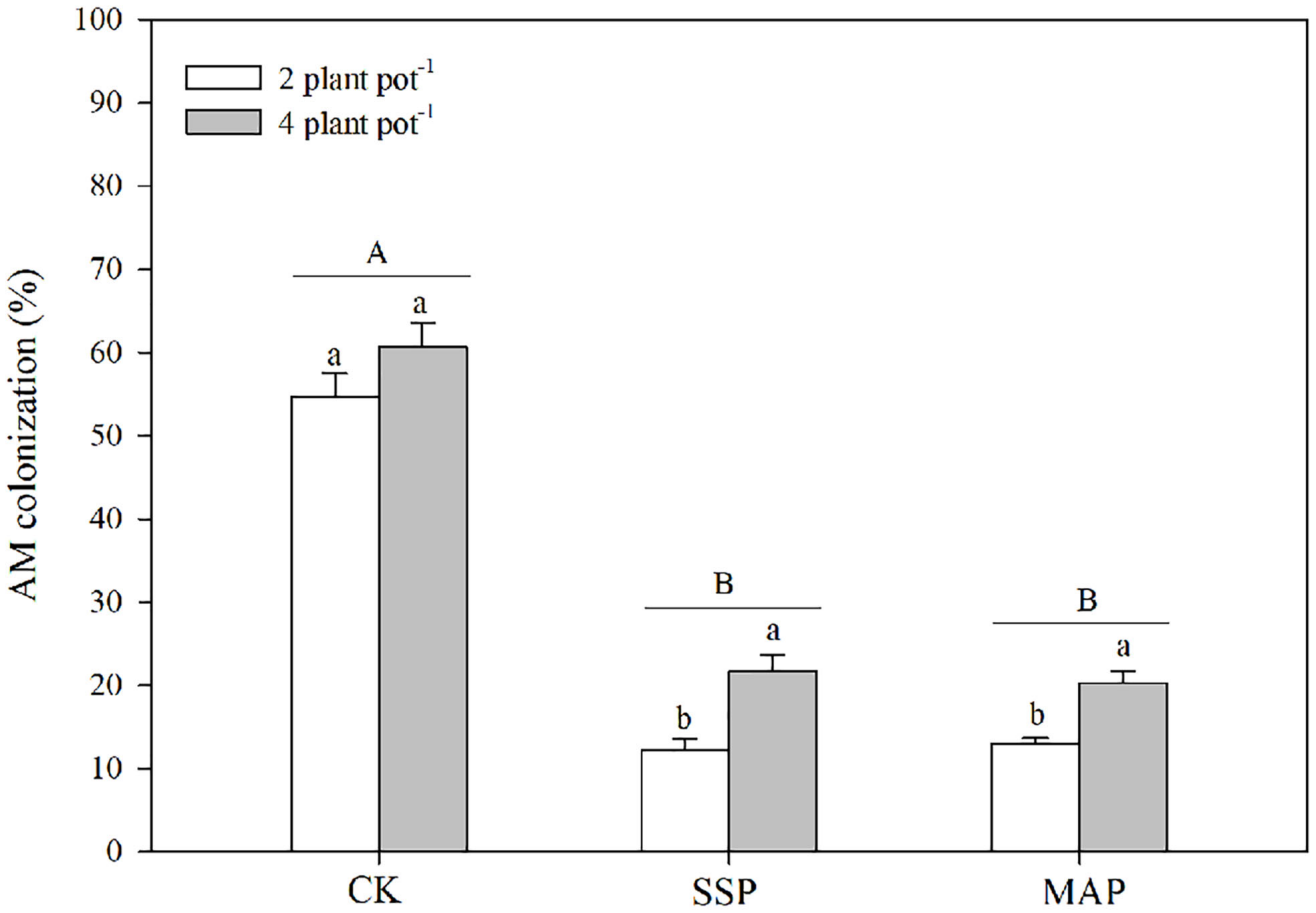
Arbuscular mycorrhizal (AM) colonization under two planting densities and three P fertilizer sources, no P (CK), superphosphate (SSP), and monoammonium phosphate (MAP). Different lowercase letters represent a significant difference among different planting densities at the *P* < 0.05 level, and different uppercase letters represent significant differences among different P fertilizer sources at the *P* < 0.05 level; ns indicates no significant difference at the 95% confidence interval between the two treatments.

The arbuscular mycorrhizal (AM) colonization was significantly altered by the P supply and planting densities (**Table 1**; **Figure 4**). Specifically, AM colonization decreased with P supply regardless of the planting density. The effect of AM colonization at the high planting density was greater than that at the low planting density in both SSP and MAP treatments. However, there was no significant difference in AM colonization between the high planting density under the SSP treatment and the low planting density under the CK treatment.

## Discussion

Several studies have investigated the effects of P supply or planting density on maize growth (Zhang et al., 2019; Gong et al., 2021). This study confirmed prior findings by demonstrating strong interaction effects between P supply and planting density. However, little is known about whether the negative effects of low planting density on shoot biomass accumulation and P uptake can be offset by increasing P supply. P plays an important role, and aboveground biomass accumulations are a response to P availability (Cheng et al., 2020; Nkrumah et al., 2021). Shoot biomass accumulation and P uptake can be increased by intensifying the P supply or changing the P source at low planting density. Similar levels of root biomass accumulation were observed at a low planting density under SSP conditions and at a high planting density under the CK treatment. These findings suggest that limited aboveground dry-matter accumulations in maize caused by low planting density are compensated for by increasing P supply intensity. It is unclear whether substituting MAP for SSP can compensate for a low shoot biomass accumulation and P uptake caused by a low planting density. The SSP treatment resulted in a high shoot biomass accumulation and P uptake at a high planting density, which were equivalent to those at the low density under MAP treatment. These findings indicate that limited aboveground dry-matter accumulations in maize at low planting densities could be compensated for by substituting MAP for SSP.

Low planting density reduces the leaf area index and the interception of photosynthetically active radiation by the canopy, resulting in a decrease in the accumulation of aboveground dry-matter (Zhang et al., 2021). Consistently, we found that at low planting densities, shoot biomass accumulation and P uptake were lower than at high planting densities. This finding in the present study was similar to those in previous studies (Du et al., 2021; Gong et al., 2021). P supply may affect root architecture and morphology, and could facilitate the establishment of a better root system (Williamson et al., 2001). It may also affect P uptake by maize plants (Hopkins and Hansen, 2019; Pongrac et al., 2020). In this study on maize, root architecture, root biomass, root length, root/shoot ratio, and lateral root density were all significantly higher under P supply than under the CK treatment. Consistent with a previous study (Zhang et al., 2017), AM colonization decreased when P supply intensity increased. A high P supply intensity may have contributed to the selection of less beneficial AM species (Saia et al., 2015). These changes in maize roots effectively increased the amount of P accessible to maize plants, supporting previous findings (Gong et al., 2021). Root growth may explain the complementary effects of P supply and planting density on shoot biomass and P uptake (Zhang et al., 2014; Gong et al., 2021). The proportion of labile P and the moderately labile P content in the SSP treatment was higher than that in the CK treatment in both planting densities tested. Labile P and moderately labile P contents were transformed into other more available forms of P for plant uptake under the SSP treatment at low planting densities, compared with that under the CK treatment at high planting densities. Our study provides substantial evidence for the complementary effects of P supply intensity and planting density on shoot biomass and P uptake.

Increasing planting density is an effective method for improving output yield in agricultural systems (Li et al., 2021; Zhou et al., 2019b). A similar shoot biomass accumulation and P uptake were observed for the same P supply intensity at a high planting density under the SSP treatment and at a low planting density under the MAP treatment. Furthermore, the values of maize root-architecture components, such as root biomass, root length, root/shoot ratio, and lateral root density, were equivalent under the MAP treatment combined with a low planting density to those observed under the SSP treatment combined with a high planting density. MAP could provide readily available P to plants, and contribute indirectly to improved root growth and development because of the beneficial starter effects of MAP (Chien et al., 2011; Gong et al., 2022) because MAP is produced from ammonia and phosphoric acid, and phosphate ( ${\text{PO}}_{4}^{3-}$ ) and ammonium (${\text{NH}}_{4}^{+}$ ) are released upon dissolution. ${\text{NH}}_{4}^{+}$ and ${\text{PO}}_{4}^{3-}$ ions are macronutrients that enhance root elongation (Weligama et al., 2008; Lu et al., 2019). When MAP was applied, shoot biomass and P uptake were higher than when SSP was applied at the same planting density. Low shoot biomass accumulation and P uptake due to low planting density may be compensated for by substituting MAP for SSP through exploiting the biological potential for enhancing P use efficiency. This implies a compensatory effect between P source and planting density.

## Conclusions

In this study, P supply and planting density significantly modified root morphology and soil P dynamics, thereby influencing shoot biomass accumulation and P uptake. A similar shoot biomass accumulation was observed at a low planting density under SSP treatment and at a high planting density under CK treatment, with similar trends in P uptake, root morphological traits, and AM colonization. The reduction in aboveground dry-matter accumulation was fully compensated for by P supply at a low planting density. Additionally, at the same P supply level, shoot biomass accumulation and P uptake at a high planting density under SSP were equivalent to those observed at a low planting density under the MAP treatment, indicating that the reduction of aboveground dry-matter accumulation in the low planting density was fully compensated for by substituting MAP for SSP. These results contribute to our understanding of the biological potentials that can be exploited to enhance P use efficiency by optimizing the types and intensities of P fertilizer. Furthermore, our study provides new insights into the compensatory effects of P supply and planting density on maize production.

## Data availability statement

The original contributions presented in the study are included in the article/**Supplementary Material**. Further inquiries can be directed to the corresponding author.

## Author contributions

XJ conceived and designed the experiments. YX, BK, and HG performed the experiments. HG analyzed the data and wrote the manuscript. All authors reviewed and approved the manuscript for publication.

## Funding

This work was supported by the National Natural Science Foundation of China (NSFC) (32172675) and by the Deutsche Forschungsgemeinschaft (DFG, German Research Foundation)-328017493/GRK 2366 (Sino-German International Research Training Group AMAIZE-P).

## Conflict of interest

The authors declare that the research was conducted in the absence of any commercial or financial relationships that could be construed as a potential conflict of interest.

## Publisher’s note

All claims expressed in this article are solely those of the authors and do not necessarily represent those of their affiliated organizations, or those of the publisher, the editors and the reviewers. Any product that may be evaluated in this article, or claim that may be made by its manufacturer, is not guaranteed or endorsed by the publisher.
